# Chronic low back pain, Modic changes and low-grade virulent infection: efficacy of antibiotic treatment

**DOI:** 10.2144/fsoa-2021-0026

**Published:** 2021-04-07

**Authors:** Claus Manniche, Gerard M Hall

**Affiliations:** 1 Department of Occupational & Environmental Medicine, Odense University Hospital, Institute of Clinical Research, University of Southern Denmark, Odense, Denmark; 2 Princess Grace Hospital, London, UK

**Keywords:** antibiotics, *Cutibacterium acnes*, disc, infection, low back pain, Modic changes

## Abstract

Chronic low back pain (CLBP) has consistently been associated with the longest number of years lived with a disability in global studies, while commonly used treatments for CLBP are largely ineffective. In 2013 a randomized, double-blind, controlled study demonstrated significant improvements in CLBP patients demonstrating Modic changes type 1 on their MRI scans and undergoing long-term oral antibiotic treatment (100 days). Much of the ensuing debate has focused on whether this was a true infection or contamination. Newer and more advanced technologies clearly point to an ongoing low-grade infection. We have reviewed all of the clinical trials published in the recent past and conclude that there is compelling evidence for the effect of long-term oral antibiotic treatment for this patient group.

## Lay abstract

Long-term oral antibiotic therapy has been shown to be effective for a subgroup of patients experiencing chronic low back pain and with Modic changes seen on their MRI scans. Newer and more sophisticated analyses have demonstrated that these Modic changes are often due to an ongoing low-grade infection. We provide an update of treatment results from the published literature and conclude that the evidence for the effectiveness of this new therapy is compelling and that further studies should focus on identifying which patients are best suited to undergo this form of treatment.

Chronic low back pain (CLBP) affects up to one billion people around the world, according to the Global Burden of Disease study of 2015, and has consistently been one of the major global causes of ‘years living with disability’ [1]. Worldwide, the use of unfounded, ineffective and costly diagnostics and treatments is widespread – and treatments may be inappropriately repeated if not effective the first time [2,3].

The development of edema in one or more vertebral bones – observed on MRI scans as Modic changes (MC) – is now documented as being associated with many treatment-resistant pain episodes and may result in patients entering CLBP status; furthermore, it is an independent prognostic marker for poor clinical outcomes [4–8]. In CLBP patients the prevalence of MC has been reported to be approximately 30–40%, as opposed to 6% in the adult background population [9,10].

The literature describes two common types of MC in the vertebral bone: type 1 and type 2. MC type 1 is characterized by high signal intensity on T2-weighted MRI scans and low signal intensity on T1-weighted scans. MC type 2 presents with relatively high signal intensity on both T1- and T2-weighted MRI [11–13].

Clinically, MC are often associated with constant lower back pain at all times during the day as well as nightly pain and morning pain/stiffness [14,15]. The literature has described a correlation over time between the extent of the MC volume and the reported pain intensity [16].

Bone biopsy studies demonstrate that MC type 1 contains a relatively high concentration of active inflammatory mediators, while MC type 2 consists of granulation tissue mixed with yellow fatty tissue and has lower levels of inflammatory activity [11,17]. A positive correlation between MC type 1 and prognosis – not seen in type 2 – has been demonstrated and this is most likely due to the greater level of immunological activity [18].

## Bacteria in the discs

During the last decade, international research has demonstrated that low-virulent intradiscal infections in some patients may trigger a progressive disc degeneration and the development of MC [19]. Newer, compelling data supporting the presence of discal bacterial infection have also been published through the last decade by several microbiological researchers from studies involving both animals and humans [20–24].

A very recently published review by Gilligan *et al.* looked closely at 19 published microbiological studies that investigated the presence of *Cutibacterium acnes* (previously called *Propionibacterium acnes*) in human intervertebral discs [25]. Varying methods to identify the bacteria were employed in these studies, leading to differing findings. The review concluded that *C. acnes* bacteria were found in 12 studies, while seven studies were only able to identify traces of the bacteria; these were interpreted to be incidental or the result of contamination from the skin. It is noteworthy that only two of the 19 studies employed proper and up-to-date quantitative microbiological techniques to adequately identify *C. acnes* infection and both of these studies found positive signs of *C. acnes* infection [26].

In tandem with the studies mentioned above, new histological studies [20,21] utilizing staining techniques through fluorescence *in situ* hybridization provide additional evidence for the existence of an intradiscal infectious pathway. Two independent research groups were able to visualize aggregates of *in situ* bacteria dispersed within disc tissue as well as associated inflammatory cells. These findings support a true and ongoing infection causing a significant immunological response within the disc. The bacterial aggregates were dispersed throughout the disc, sometimes with significant distance between them. This is the likely reason why traditional microbiological techniques are insufficient for the detection of these bacteria as opposed to classical bacterial infection, due to the relatively low number of bacteria observed in low-grade infections.

Microbiological studies of the past 10 years, combined with new imaging technologies, have led to a better understanding of the now definitively demonstrated infectious pathway leading to MC and CLBP [8,25]. This knowledge should inspire researchers to carry out further studies as regards both diagnostics and treatment. Researchers in this field who previously have expressed skepticism regarding an infectious pathway may consider re-evaluating their positions; the evidence is now beyond reasonable dispute [27,28].

## Clinical antibiotic trials

The disc infection hypothesis has led to a number of clinical trials carried out by independent research groups using per-oral antibiotics to treat patients with CLBP and MRI-proven MC. In this paper we will conduct an update and review of these trials and present a summary of the results based on the methodology, outcomes and side effects reported in each study. We include published randomized controlled trials (RCTs) as well as open-label studies published between 2005 and 2020 in the update.

We have identified two high-quality RCTs [29,30], a recently published *post-hoc* subgroup analysis [31], a smaller RCT which did not include follow-up data [32] and seven additional open-label studies, several of which had incomplete datasets [33–39].

In the Albert *et al.*study [29], 162 patients were randomized to receive treatment, which consisted of amoxicillin–clavulanate (Bioclavid; 1500/375 mg or 3000/750 mg daily) for 100 days or placebo tablets. There were no significant differences between groups at baseline, and 90.7% of patients completed the 12-month follow-up questionnaires. All of the main outcome measures demonstrated strong improvements and were statistically significant. In the actively treated group, the Roland–Morris Disability Questionnaire (RMDQ) improved by 53.4%, back pain by 44.8% and leg pain by 68%, whereas the placebo group did not demonstrate any improvement in any end points. The number of hours with pain in a 4-week period (maximum 448 h) fell from 448 to 64 h in the actively treated group, while no reduction was observed in the placebo group at 12-month follow-up. Adverse events were more common in the antibiotic group (65% of participants) than the placebo group (23%). These were mainly low-grade gastroenterological complaints such as increased flatulence or burping. Follow-up MRI scans at 1 year revealed a significant decrease in MC volume in the antibiotic group, while no change was seen in the placebo group (p = 0.05).

The Bråten *et al.* study [30] included both patients with MC1 (n = 118) and those with MC2 (n = 62), and the results were primarily presented as mixed MC1 and MC2. Patients were randomized to 3 months of oral treatment with either 2250 mg amoxicillin daily or placebo tablets. We present the data regarding the MC1 patients only, because patients with MC2 represent a different state of the condition and furthermore did less well than even the placebo group. The primary outcome measure was designated as the RMDQ-24. A statistically significant difference can be seen in all of the outcome measures except back pain (p = 0.06).

In the amoxicillin group, 50 patients (56%) had at least one drug-related adverse event; 31 patients (34%) in the placebo group also reported adverse events. One or more serious adverse events occurred in six patients (7%) in the amoxicillin group and two patients (2%) in the placebo group; none was related to the study drug. In the amoxicillin group, 11 patients (12%) discontinued or paused the study drug due to adverse events, compared with two patients (2%) in the placebo group.

Table 1 includes comparative data between the two studies.

**Table 1.** A comparison of outcome measures at baseline and 1-year follow-up for patients with Modic changes type 1 in the Albert and Bråten trials.

	Albert *et al.*, Modic type 1, n = 162^†^					Bråten *et al.*, Modic type 1, n = 118^‡^					
	Baseline		1 year			Baseline		1 year			[29,30]
	Placebo	Antibiotic	Placebo	Antibiotic	p-value	Placebo	Antibiotic	Placebo	Antibiotic	p-value	
RMDQ score	15.0 (12;18)	15.0 (11;18)	14.0 (11;18)	7 (4;11)	0.0001	12.3 (3.7)	12.9 (4.3)	10.3 (5.4)	8.2 (6.0)	0.02	
LBP score	6.3 (4.7;8)	6.7 (5.3;7.0)	6.3 (4;7.7)	3.7 (1.3;5:8)	0.0004	6.3 (1.3)	6.5 (1.1)	5.2 (2.2)	4.5 (2.5)	0.06	
EQ-5D score	60 (40;75)	59 (40;70)	60 (40;75)	75 (54;90)	0.0014	56 (0.16)	55 (0.18)	60 (0.21)	66 (0.21)	0.03	

† Values are presented as median and lower; upper quartiles.

‡ Values presented as mean and standard deviations.

EQ-5D: EuroQol- 5 Dimension; LBP: Lower back pain; RMDQ: Roland–Morris Disability Questionnaire.

Kristoffersen *et al.* [31] recently published a subgroup analysis of patients with short TI inversion recovery (STIR) sequences as seen on MRI who were enrolled in the Bråten *et al.* study [30] in order to access whether patients with high signal intensity as seen on STIR sequences (MC1) reacted to oral antibiotic treatment when adjusting outcomes by volume, height or maximal intensity of signal increase on both sides of the disc. As predicted, patients fulfilling all of the above criteria (STIR3) improved their disability status by -5.1 RMDQ points (95% CI: -8.2 to -1.9; p for interaction = 0.008) compared with placebo patients. For the STIR2 group, volume ≥25% groups, the effect of amoxicillin was to alter the score by >4 RMDQ points (the cutoff for clinical importance), but not for lower back pain intensity. Among patients receiving amoxicillin, six of 22 STIR3 patients (27%) improved by >75% compared with nine of 41 (22%) STIR2 patients. Patients with volumes of <10% (STIR1) did less well.

The smaller RCT conducted by Al-Falahi *et al.* included 71 patients with CLBP and MC1 changes [32]. They were treated with amoxicillin–clavulanate (Bioclavid; 1000/250 mg) daily for 100 days or placebo tablets. Forty-three patients (60%) completed the study. Follow-up results at 3 months demonstrated a statistically and clinically significant difference favoring the antibiotic group for both pain and disability; the relative improvements were extremely similar to those found by Albert *et al.* [29]. No improvements were noted in the placebo group. Mild adverse events were more common in the antibiotic group (60% of participants) compared with the placebo group (6%). Considerable side effects were noted in 4 and 3% of participants, respectively.

## Results for small studies

An overview of these trials can be seen in Table 2.

**Table 2.** An overview of the nonrandomized studies.

Study (year)	n	MC1/MC2	Drop-outs	Primary outcome parameter	Follow-up (months)	Adverse effect report	Ref.
Albert *et al.* (2008)	32	MC1	3	RMDQ23	12/12	Yes	[33]
Albert *et al.* (2017)	1024	MC1 or MC2	37	RMDQ23	24/12	No	[34]
Hammond *et al.* (2015)	30	NS	8	RMDQ23	3/12	Yes	[35]
Manniche *et al.* (2016)	147	MC1	0	Patients Global Assessment	6/12	Yes	[36]
Palazzo *et al.* (2017)	28	MC1	8	Patients Global Assessment	12/12	Yes	[37]
Schepers *et al.* (2019)	30	NS	0	RMDQ	12/12	Yes	[38]
Gupta *et al.*(2017)	11	NS	NS	Pain, Numeric Rating Scale	11/11	NS	[39]

MC: Modic changes; NS: Not stated; RMDQ: Roland–Morris disability questionnaire.

In a pilot study, 32 consecutive patients with MC1 were treated with amoxicillin–clavulanate (Bioclavid; 1500/375 mg) daily for 90 days [33]. Data for 29 patients were available at the end of treatment and follow-up (mean of 10.8 months); 62% of patients reported that they had improved their disability scores (RMDQ) by 30% or more at the end of treatment and retained their improvement at end follow-up. Three patients dropped out of the study due to severe diarrhea.

A large ongoing open-label study [34] including 1024 consecutive patients with MC1, MC2 and mixed MC1/MC2 treated with amoxicillin (3000 mg daily) for 100 days demonstrated strikingly similar results to the original Albert *et al.* and Al-Falahi *et al.* trials [29,32]. As this trial is still ongoing, data are currently only available for 602 (59%) of patients at a 12-month follow-up. It is noteworthy that patients continue to improve from 1-year to 2-year follow-up.

An open-label study of 33 consecutive patients, of whom 22 completed the end-of-treatment questionnaires, was carried out by Hammond [35]. 40.9% rated their improvement as excellent (≥75%), 27.3% as good (≥50%) and 6.7% as moderate (≥25%). No major adverse events were recorded.

Manniche *et al.* [36] studied a hospital outpatient cohort including 147 CLBP patients with MC1 who had undergone several other types of treatments without success, to evaluate the effect of treatment with amoxicillin–clavulanate (Bioclavid; 1500 mg/375 mg) daily for 3 months. At 6-month follow-up, 78/147 (53%) of the patients stated that they had a positive response. At 6 months, pain in the responder group fell by 30% compared with 2% in the nonresponder group. Overall, adverse effects were observed in 40% of the patients.

In a published letter describing a cohort of patients treated with antibiotics, 28 patients were included, of whom eight did not complete the treatment [37]. The dataset is incomplete. Pain reduction was calculated to be statistically significant (p = 0.048) at 12-month follow-up, as was night-time awakening (p = 0.02). Six patients improved their global perceived effect by ≥50% and four patients by ≥70% at 12 months. Four patients reported severe side effects, while 11 (39.3%) reported side effects of a less severe nature.

A stepped care model involving CLBP patients with MC1 (n = 106) was employed in another study [38]. Patients were consecutively chosen who had not experienced benefit from conservative care; they were prescribed 2 weeks of oral celecoxib (400 mg daily). Intradiscal steroid injections were administered to poor responders. If steroid injections were unsuccessful, amoxicillin (3000 mg daily) was prescribed if patients scored more than 5/10 on their numerical pain scores. 60% of patients undergoing antibiotic treatment improved their pain scores by ≥2 points and their RMDQ by 3 points; 43% improved their pain scores by 50% and their RMDQ by 40%. No treatment complications were reported.

Lastly, a small (n = 11) retrospective case series analysis was carried out by Gupta [39]. The antibiotic dosage was amoxicillin–clavulanate (Bioclavid: 1500/375 mg per day) for 3 months. The dataset is incomplete. The authors concluded that the use of antibiotics in patients with Modic changes and lower back pain had limited efficacy. Adverse effects were low-grade gastrointestinal complaints such as loose bowel movements, increased flatus or burping, with less than 3% of patients discontinuing therapy because of drug-related adverse reactions.

This series of trials (n = 8) includes a small RCT, a large open-label study, an audit from a university hospital and several independently conducted trials on patients who did not respond to conservative care. While bearing in mind the significant limitations associated with the interpretation of open-label trial results, there nonetheless appears to be a positive overall effect of oral antibiotic treatment in at least 50% of patients with MC1.

## Discussion

The earlier uncertainty surrounding a possible low-grade infectious pathway leading to disc degeneration, endplate disruption and vertebral edema reflecting a low-grade bacterial infection as opposed to contamination was founded on a large number of studies of varying quality. However, newer studies that utilize more sophisticated technologies should have laid the ‘contamination versus infection’ debate to rest [25]. Bacterial burdens that are too high to be due to contamination have been published [40], and fluorescence *in situ* hybridization studies have identified bacterial aggregates, biofilms, polymorphonuclear leukocytes and inflammatory cells that leave no doubt regarding the presence of an ongoing infection [20,21].

In contrast to MC2, type 1 MC demonstrated on MRI have been shown to be predictive of poor long-term outcomes and nonresponse to conservative care. MC1 correlate strongly to current low back pain and are characterized by constant pain, nightly pain and morning stiffness compared with patients with MC2 [8,14,15]. This inspired a Danish group to carry out a high-quality RCT [30] including 162 patients with MC1 changes and CLBP who were treated with either amoxicillin–clavulanate for 100 days or placebo tablets. A battery of outcome measures all demonstrated statistically and clinically significant improvements at the end of the intervention and further improvement at the 12-month follow-up. The publication of this trial received considerable interest when it was published in 2013, as it represented a potential paradigm shift for a significant group of treatment-resistant CLBP patients with MC1 changes. Previous treatment for these patients has been largely ineffective [41,42].

A second high-quality RCT was published in 2019 [30]. The authors described their study as a replication of that carried out by Albert *et al.* in 2013 [29], even though a different antibiotic regime was used, as well as another version of the RMDQ. Additionally, the authors included patients with both MC1 and MC2, as opposed to MC1 patients only in the Albert *et al.* trial [29]. They wrote: *“In contrast to the trial we were reassessing, we chose to include patients with type 2 Modic changes because differentiating between type 1 and type 2 Modic changes is of uncertain relevance…”* Many of the nine reviewers questioned mixing the data of the MC1 and MC2 patients as well as questioning the authors’ main conclusions [43]. Despite statistically significant differences only being seen in MC1 patients (who improved by 36.5% on the RMDQ-24 scale, the primary outcome measure – a result clearly superior to the recommended Standard of Care Treatments for CLBP [41,42]), the main conclusion was that: *“Our results do not support the use of antibiotic treatment for chronic low back pain and Modic changes”*. A more appropriate conclusion might have been: our modified protocol demonstrated a positive effect on patients with MC1 but not patients with MC2, although our results were not as strong as those of Albert *et al.* More research must be undertaken before firm conclusions can be reached’. Now, 18 months later, the same Norwegian research group has published a new and important subgroup data analysis involving patients from their original study [31]. We are now presented with an entirely different set of conclusions which contradict those of the original article. A substantial subgroup of STIR2 and STIR3 patients demonstrated a clinically and statistically significant difference in the actively treated group compared with the placebo group on the primary outcome measurement, the RMDQ-24 (-5.1 RMDQ points; 95% CI: -8.2 to -1.9; p = 0.008). Improvements could be seen at 3- and 12-months follow-up. More than 25% of all actively treated MC1 patients achieved a noteworthy improvement in their functional disabilities of more than 75% – a result not seen with commonly recommended treatments, including spinal fusion [41,42]. The number needed to treat in this subgroup was calculated to be 3.1. The research methods of Bråten *et al.* have been previously criticized by spinal research experts for reaching generalized conclusions which were not supported by their own data [44,45]. Recently, Gilligan *et al.* [25] listed a series of methodological weaknesses and errors contained in the Bråten *et al.* study in an invited review. The positive results seen in the Albert *et al.* [29] and Bråten *et al.* [30] trials are largely supported by the open-label studies reviewed in this paper involving patients with MC1 and CLBP.

Unfortunately, contradictory conclusions and an oscillating focus between MC1 and MC2 have created a predictable degree of confusion among researchers and clinicians that could hinder further research regarding antibiotic treatment of MC1 patients with CLBP, thus misleading the scientific community as well as preventing carefully selected patients from receiving appropriate care.

## Conclusion

New compelling microbiological studies of the past 5 years, combined with enhanced imaging technologies, have led to a better understanding of the now definitively demonstrated infectious pathway leading to MC and CLBP. This update review included two high-quality RCTs, one small RCT without follow-up data, a *post hoc* subgroup analysis and several open-label studies of varying sizes. All three RCTs demonstrated statistically significant results for patients with CLBP and MC1 on their MRI scans after undergoing long-term oral antibiotic treatment. There does not appear to be the same effect with CLBP patients demonstrating MC2 on their MRI scans. The subgroup analysis identified a substantial group of patients that achieved what can only be described as extraordinary results.

## Future perspective

Additional trials should be undertaken to further validate the usefulness of long-term oral antibiotic treatment with a focus on patient selection. Other means of delivering antibiotics to the site of infection should also be investigated. Work relating to the development of biomarkers would be most welcome as the percentage of patients with MC1 changes that are due to infection has not been firmly determined, and it would be most helpful in clinical practice to be able to distinguish between MC due to infection and those caused by a mechanically induced autoimmune response.

**Executive summary**

- Modic changes (MC) are now documented as being involved in many treatment-resistant chronic lower back pain episodes.
- Clinically, MC are often associated with constant lower back pain at all times during the day as well as nightly pain.
- Newer and more sophisticated analyses have demonstrated that MC are often associated with an ongoing low-grade infection.
- Three randomized controlled trials demonstrated statistically significant and clinically meaningful results for patients with chronic lower back pain and MC type 1 on their MRI scans after undergoing long-term oral antibiotic treatment.
- A subgroup analysis identified a substantial group of patients who achieved a resolution of their infection.
- Patients with MC type 2 do not appear to derive the same benefit from antibiotic treatment as patients with MC type 1.
